# Performance enhancement of asymmetric supercapacitors with bud-like Cu-doped Mn_3_O_4_ hollow and porous structures on nickel foam as positive electrodes†

**DOI:** 10.1039/c8ra06989a

**Published:** 2018-10-22

**Authors:** Xiaobo Chen, Cheng Chen, Tianzhi Xu, Yingjie Xu, Weiwei Liu, Wen Yang, Peizhi Yang

**Affiliations:** School of New Energy and Electronic Engineering, Yancheng Teachers University Yancheng 224051 PR China; Key Laboratory of Education Ministry for Advance Technique and Preparation of Renewable Energy Materials, Yunnan Normal University Kunming 650500 PR China pzhyang@hotmail.com

## Abstract

Cu-doped Mn_3_O_4_ hollow nanostructures supported on Ni foams as high-performance electrode materials for supercapacitors were successfully synthesized through a facile hydrothermal method and subsequent calcination. The morphology, structure, and electrochemical performance of the as-prepared Mn_3_O_4_ nanostructures can be tuned just by varying the Cu doping content. Benefiting from the unique bud-like hollow structure, the 1.5 at% Cu-doped Mn_3_O_4_ sample has a high specific capacitance of 257.6 F g^−1^ at 1 A g^−1^ and remarkable stability (about 90.6% retention of its initial capacitance after 6000 electrochemical cycles). Besides, an asymmetric supercapacitor (ASC) cell based on the 1.5 at% Cu-doped Mn_3_O_4_ exhibits a high specific capacitance of 305.6 F g^−1^ at 1 A g^−1^ and an energy density of 108.6 W h kg^−1^ at a power density of 799.9 W kg^−1^. More importantly, the ASC shows good long-term stability with 86.9% capacity retention after charging/discharging for 6000 cycles at a high current density of 5 A g^−1^.

## Abstract 2

The effect of Cu doping on the electrochemical performance of bud-like Mn_3_O_4_ nanostructures for supercapacitor application was comparatively investigated.

## Graphical abstract

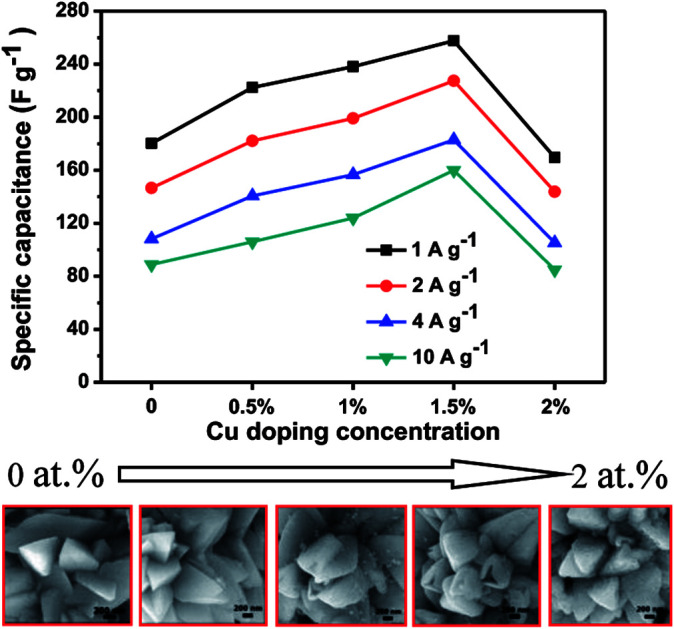

## 1. Introduction

In recent years, supercapacitors (SCs) have been widely recognized in a wide range of energy storage applications due to their properties such as their faster charging–discharging, higher specific power density, and longer cycling life compared to batteries.^1,2^ In particular, asymmetric supercapacitors (ASCs), which usually include a faradic electrode (as energy source) and a capacitive electrode (as power source) in one system, have been considered a promising strategy to increase the potential window and the energy density.^3^ The capacitive electrode materials generally involve various metal oxides,^4–6^ metal hydroxides,^7,8^ metal sulphides,^9,10^ metal carbonate hydroxides^11,12^ and conductive polymers.^7,13^ Among these materials, hausmannite (Mn_3_O_4_) has shown promising potential in the field of supercapacitors on account of its low-cost, low toxicity, environmental benignity^14^ and satisfactory supercapacitive performance.^15–17^ Compared with MnO_2_, Mn_3_O_4_ has much superior properties, such as uniform structure, stable performance and easily obtained pristine phase Mn_3_O_4_.^18^

However, the direct application of pure Mn_3_O_4_ in supercapacitors is limited owing to its low electronic conductivity, low specific capacitance values and non-ideal rate performances.^7^ Doping of various elements such as Sn,^19^ Cr,^2^ Co^2^ and Ni^2^ into Mn_3_O_4_ compounds has been found to be effective way to promote its conductivity and capacitance. It is obvious that doping with the selective metal ions may overcome the limitations of electronic states and also generate new phenomena. Su *et al.*^20^ reported the utilization of Cu-doped δ-MnO_2_ as electrode material for a supercapacitor electrode, and exhibited higher capacitance and better cycle performance than that of the undoped samples. To our best knowledge, we have also not found the report on the preparation of Cu-doped Mn_3_O_4_ as electrode materials for supercapacitors.^2^ Moreover, the capacitive behavior of Mn_3_O_4_ electrode critically depends on its crystal structure and morphology.^16^ Therefore, the majority of studies on Mn_3_O_4_ electrode material for supercapacitors have focused on achieving the highest capacitance by adjusting growth conditions to obtain Mn_3_O_4_ with desirable morphologies. These morphologies include nanospheres,^21^ nanorods,^22^ nanosheets,^23^ and nanofibers.^24^ The unique bud-like morphology of Mn_3_O_4_ will be reported in this paper.

In this work, we explore the effect of Cu doping on the supercapacitor performance of Mn_3_O_4_-based electrodes, synthesized by a one-pot hydrothermal method. The effects of Cu doping on the modification of structure, surface morphology and electrochemical performance have been studied exhaustively and discussed in detail.

## 2. Experimental

All chemicals reagents were of analytical-reagent grade and purchased from Zhanyun Chemical Reagent Co., Ltd (Shanghai, China) without further purification. Prior to use, the fresh Ni foam was slightly etched in an ultrasonic bath with 3 M HCl to remove oxides, and then washed thoroughly with deionized water and ethanol respectively. The Mn_3_O_4_ (or Cu-doped Mn_3_O_4_) samples were obtained by thermally annealing MnCO_3_ (or Cu-doped MnCO_3_) precursors in the presence of nitrogen gas flow, and the MnCO_3_ (or Cu-doped MnCO_3_) precursor was synthesized by a modified procedure according to reference.^25^ Mn(CH_3_COO)_2_·4H_2_O (3.48 g), urea (CO(NH_2_)_2_) (5.07 g) and of NH_4_F (1.63 g) were dissolved one by one in 10 min interval with constant stirring in 70 mL of ethanol solution to form a homogenous solution. Doping concentration of cupric nitrate (Cu(NO_3_)_2_·6H_2_O) was 0, 0.5, 1, 1.5 and 2 at%, which refers to the molar ratio of Cu to Mn in the reactants. The total volume was then brought up to 350 mL with distilled water and transferred into ten 50 mL Teflon-lined autoclaves with 8 pieces of cleaned Ni foams (1 × 1 cm^2^ in sizes) in each autoclave. The autoclave was kept at 160 °C for 16 h in an electric oven and cooled down to room temperature. Finally, the precursors grown Ni foam were collected by washing with distilled water and ethanol, then dried in a vacuum furnace at 60 °C for 6 h and further calcined in nitrogen gas at 400 °C for 3 h. The total mass loading on nickel foam was around 2 mg cm^−2^ for all the five fabricated electrodes.

The crystalline structures of the samples was characterized by X-ray diffraction (XRD) with use of a Smartlab-9 X-ray diffractometer with Cu Kα radiation (*λ* = 1.5418 Å). The microstructure and chemical composition of the Cu-doped Mn_3_O_4_ samples was characterized by scanning electron microscopy (SEM, Zeiss Supra 35VP, USA) equipped with an energy-dispersive X-ray spectroscopy (EDS). The chemical composition of the Cu-doped Mn_3_O_4_ samples was also were determined by means of an inductively-coupled plasma (ICP) mass spectrometer (ICP-AES, Horiba Jobin Yvon). Specific surface areas were measured with a Brunauer–Emmett–Teller (BET) sorptometer (SSA-4300, Beijing Builder, Inc.) using N_2_ adsorption at 77 K. To evaluate the electrochemical performance of the fabricated electrodes, cyclic voltammetry (CV), galvanostatic charge–discharge (GCD), and electrochemical impedance spectroscopy (EIS) measurements were conducted in a three-electrode cell in N_2_-saturated 1.0 M Na_2_SO_4_ aqueous solution. The electrochemical performances were recorded on a conventional CHI660E setup comprising a saturated calomel electrode (SCE) reference electrode, a counter electrode of Pt sheet, and a working electrode of Ni foam supported prepared Mn_3_O_4_-based electrode.

## 3. Results and discussion

**Fig. 1:**
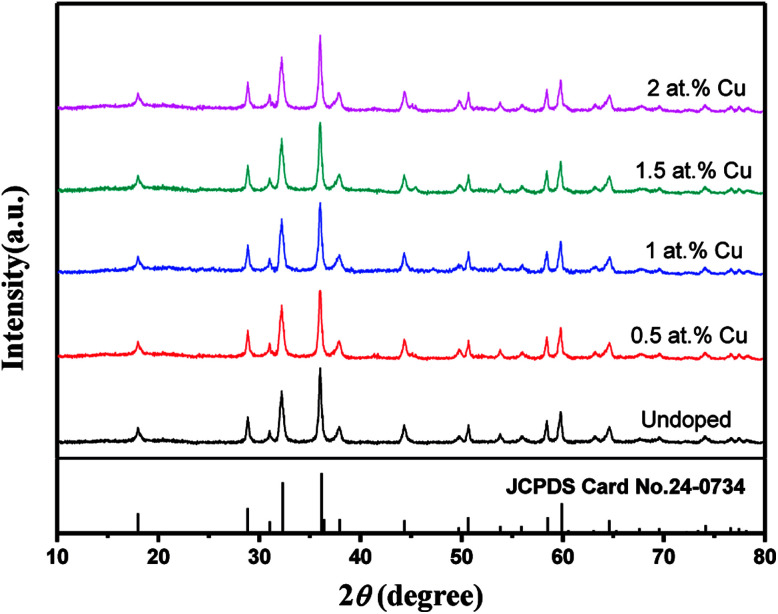
XRD patterns of undoped and Cu-doped Mn_3_O_4_ samples.

As shown in Fig. S1,† the MnCO_3_ and Cu-doped MnCO_3_ precursors with rhodochrosite structure (JCPDS card no. 44-1472) were first synthesized. Fig. 1 shows the XRD patterns of the pure Mn_3_O_4_ and Cu-doped Mn_3_O_4_ powders scraped from Ni foam. It can be seen that all samples possess similar XRD patterns and all diffraction peaks can be indexed to the hausmannite Mn_3_O_4_ (JCPDS no. 24-0734). Six dominant diffraction peaks centered at 2*θ* = 18.0°, 28.9°, 32.3°, 36.1°, 38.0°, and 58.5° can be attributed to the crystal planes of (101), (112), (103), (211), (004), (321), and (224), respectively. This means Cu dopant has no influence on the crystal structure of the Mn_3_O_4_. No diffraction peak corresponding to the Cu species is seen in all the samples, probably owing to the low content of Cu in the Mn_3_O_4_ sample or the uniform doping of Cu ion into the Mn_3_O_4_ nanoparticles.

**Fig. 2:**
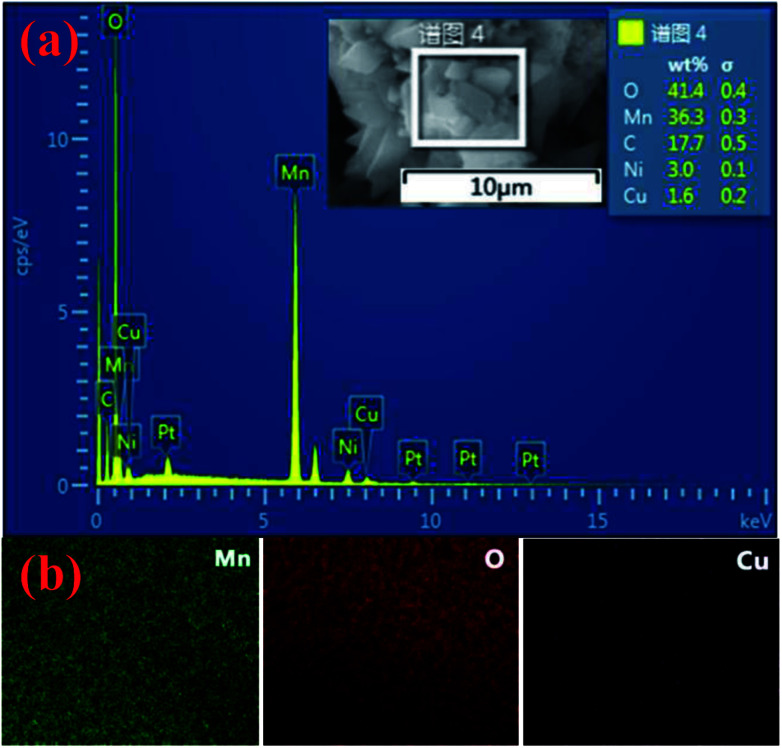
(a) Elemental mapping of Mn, Cu, and O for 1.5 at% Cu-doped Mn_3_O_4_; (b) EDS of 1.5 at% Cu-doped Mn_3_O_4_.

The Cu/Mn atomic ratios of various Cu-doped Mn_3_O_4_ were also determined by ICP spectrometer and EDS, and listed as in Table 1. The representative EDS microanalysis of 1.5 at% Cu-doped Mn_3_O_4_ is shown in Fig. 2(a), which also confirms that Cu dopants have been successfully introduced into the Mn_3_O_4_. The atomic ratio of Cu/Mn was found to be linearly proportional to the concentration of Cu in the initial reaction solution. The elemental mapping of 1.5 at% Cu-doped Mn_3_O_4_ are shown in Fig. 2(b), which intuitively shows the distribution of Mn, Cu, O. It is suggested that the Cu is uniformly distributed in the sample.

**Table 1:** The Cu/Mn atomic ratios obtained from ICP and EDS characterizations

Sample	Cu/Mn atomic ratio^a^	Cu/Mn atomic ratio^b^
Undoped Mn_3_O_4_ sample	0	0
0.5 at% Cu-doped Mn_3_O_4_ sample	0.009	0.011
1 at% Cu-doped Mn_3_O_4_ sample	0.021	0.025
1.5 at% Cu-doped Mn_3_O_4_ sample	0.032	0.038
2 at% Cu-doped Mn_3_O_4_ sample	0.045	0.049

a Obtained from ICP.

b Obtained from EDS.

**Fig. 3:**
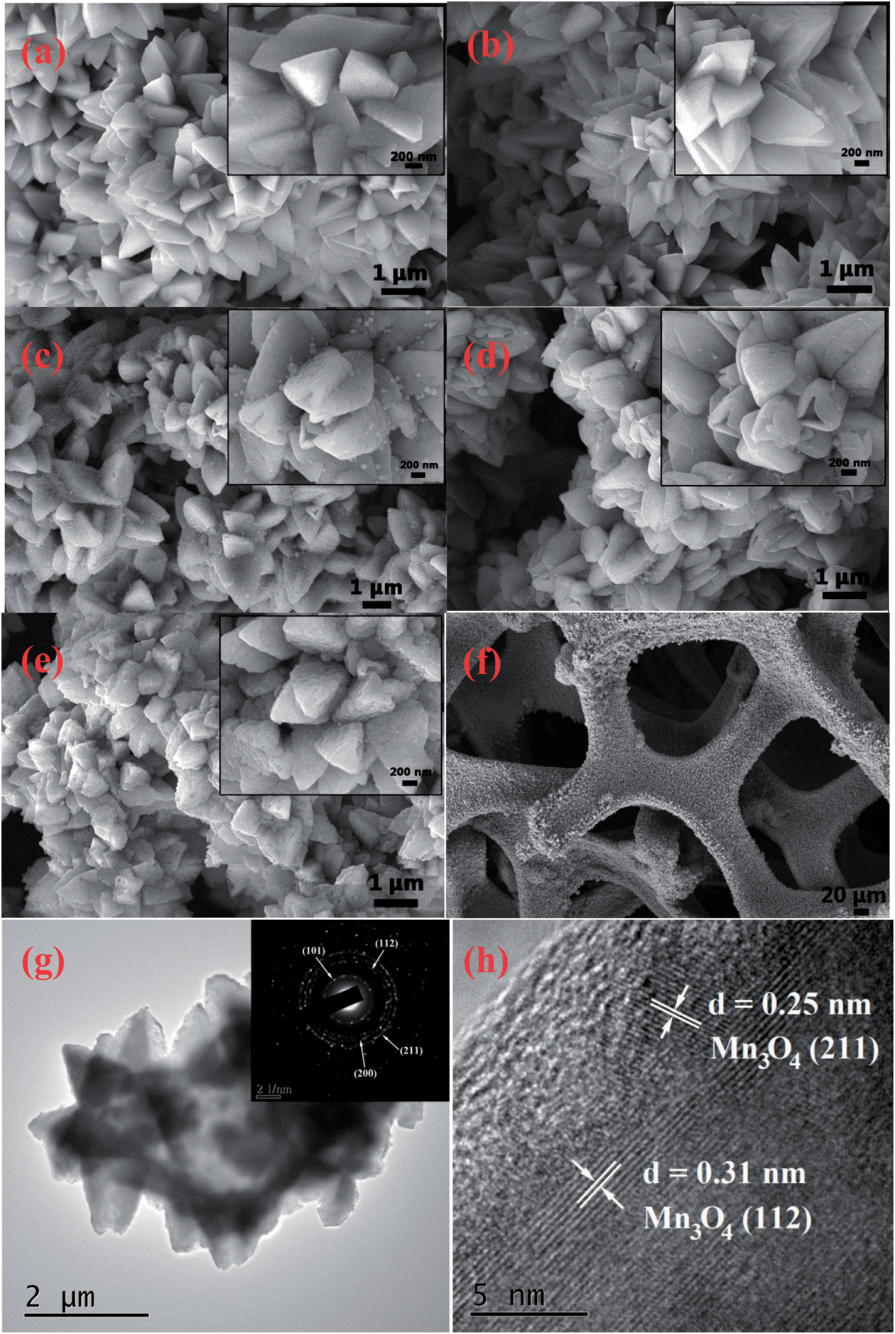
SEM images of the samples: (a) undoped, (b) 0.5 at%, (c) 1 at%, (d) 1.5 at% and (e) 2 at% Cu-doped Mn_3_O_4_ samples. (f) The 3D hierarchical structure of the 1.5 at% Cu-doped Mn_3_O_4_ grains growing on Ni foam. (g) TEM image of 1.5 at% Cu-doped Mn_3_O_4_. The inset shows the SAED pattern with indexed diffraction rings. (h) HRTEM image of 1.5 at% Cu-doped Mn_3_O_4_.

The morphologies of the samples were examined using SEM to investigate the effects of Cu doping on the nanostructure. In Fig. 3(a–e), we show the SEM images of undoped and Cu-doped Mn_3_O_4_ samples. The SEM images indicate that the Cu doping strongly affects the nanostructure of Mn_3_O_4_ sample. The insets of the SEM are the higher magnification SEM images. Fig. 3(a) shows the SEM image of the surface morphology of the undoped Mn_3_O_4_ sample. It is clearly shown that the sample consists of numerous octahedral crystals with edge lengths of nearly 1 μm. All of these crystalline grains interconnect with each other to form flower-like morphology. After 0.5 at% copper dopping, there is no obvious change in morphology. When 1 at% copper is doped into Mn_3_O_4_ sample, Fig. 3(c) indicate that a significant morphological transformation occurred. The sample shows an interesting structure composed bud-like hollow structure of Mn_3_O_4_ particles. As doping content of Cu is increased to 1.5 at%, more and more Mn_3_O_4_ grains are transformed to hollow structure. The formation of this hollow structure is assumed to take place through a self-assembly process similar to that described by Meher *et al.*^26^ in the case of MnO_2_. Therefore, the incorporation of Cu dopants may have a significant influence on the hausmannite Mn_3_O_4_, leading to the formation of hollow structure. However, as doping concentration of Cu reached 2 at%, the hollow morphology disappeared, and coarse grains were produced. As for the bud-like hollow structure of moderate Cu-doped Mn_3_O_4_ particles, it is assumed that the formation of micrometer sized hollow spheres is analogous to that reported previously.^25,27,28^ It can be explained as a fundamental solid-state phenomenon, the so-called Kirkendall effect,^27,29^ which deals with the movement of the interface between diffusion couples.

Fig. 3(f) shows a typical low-magnification SEM image of the 3D hierarchical structure of the 1.5 at% Cu-doped Mn_3_O_4_ grains growing on Ni foam. Obviously, the Mn_3_O_4_ grains are uniformly grown on the Ni foam substrate while completely maintaining the 3D grid structure of the pristine Ni foam. The XRD and SEM investigations confirm that although Cu dopant has no influence on the crystal structure the hausmannite Mn_3_O_4_, it indeed make apparent change in the morphological structure. The TEM image of the 1.5 at% Cu-doped Mn_3_O_4_ further confirms the hollow bud-like morphology (Fig. 3(g)). The SAED pattern (inset in Fig. 3(g)) of the 1.5 at% Cu-doped Mn_3_O_4_ shows a crystalline nature with the (1 0 1), (1 1 2), (2 0 0), and (2 1 1) planes of Mn_3_O_4_. Moreover, in the HRTEM image (Fig. 3(h)), the lattice fringes are clearly seen and matches well with *d*-spacing of 0.25 and 0.31 nm, corresponding to (2 1 1) and (1 1 2) planes of Mn_3_O_4_, respectively. The results are consistent with the XRD results (Fig. 1).

**Fig. 4:**
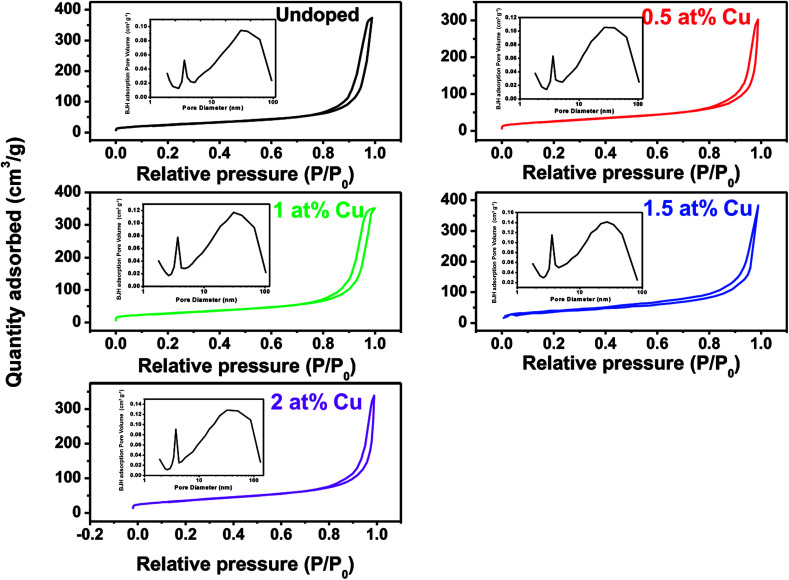
N_2_ adsorption/desorption isotherms of undoped and Cu-doped Mn_3_O_4_ samples (inset: BJH pore size distributions of undoped and Cu-doped Mn_3_O_4_ samples).

The specific surface area and pore parameters of undoped and Cu-doped Mn_3_O_4_ powders scraped from Ni foam were further investigated by measuring adsorption–desorption isotherms of nitrogen at 77 K. Fig. 4 illustrates the N_2_ adsorption/desorption isotherms and corresponding Barrett–Joyner–Halenda (BJH) adsorption pore size distribution plots (inset). According to the IUPAC classification, all these samples behave type-IV isotherms. The increase of the Cu doping concentration has a significant effect on the final textural parameters, including the surface areas and pore volume. The BET surface areas are 95.2 m^2^ g^−1^ for undoped Mn_3_O_4_, 97.8 m^2^ g^−1^ for 0.5%, 103.4 m^2^ g^−1^ for 1%, 114.5 m^2^ g^−1^ for 1.5% and 109.2 m^2^ g^−1^ for 2% Cu-doped Mn_3_O_4_, respectively. The specific pore volumes of undoped, 0.5 at%, 1 at%, 1.5 at% and 2 at% Cu-doped Mn_3_O_4_ are 248, 180, 239, 262 and 251 m^3^ g^−1^, respectively. Among the samples studied, 1.5 at% doped Mn_3_O_4_ shows the largest BET surface area and special pore volume. It is worthy of note that Cu dopant has no significant influence on the pore size of the Mn_3_O_4_ nanostructure. The pore size distribution (insets of Fig. 4) in these samples was similar, featuring pores between 2 nm and 100 nm, which mainly consisted of mesopores and macropores. These multiple mesopores pore size distribution would facilitate the electrolyte ions transfer during electrochemical charging/discharging by providing an efficient transport pathway and more adsorption sites in the interior of the electrodes,^30^ which is helpful to enhance specific capacitance of the electrodes.

**Fig. 5:**
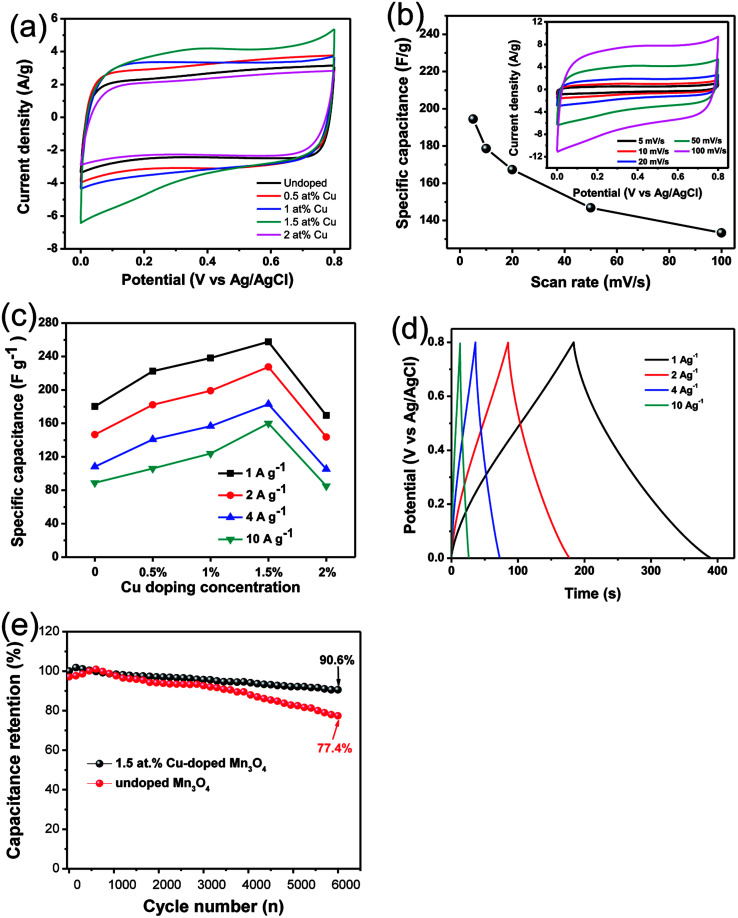
(a) CV curves of undoped and Cu-doped Mn_3_O_4_ samples at 50 mV s^−1^ and (b) CV curves of 1.5 at% Cu-doped Mn_3_O_4_ sample at different current density. (c) The plots of the specific capacitance as a function of Cu doping concentration at different scan rates. (d) Charge–discharge curve of 1.5 at% Cu-doped Mn_3_O_4_ sample. (e) Cycle performances of 1.5 at% Cu-doped Mn_3_O_4_ at the current density of 2 A g^−1^.

CV has been used to evaluate the electrochemical performances of Cu-doped Mn_3_O_4_-based electrodes (Fig. 5(a)). It is clearly illustrate that Cu dopant can affect the CV curve area. It is well known that the larger CV curve area is, the greater specific capacitance will be obtained.^31^ It can be seen that the CV curve area firstly increases and then decreases with the increase of the Cu doping concentration. It is clearly indicated that the specific capacitance of the 1.5 at% Cu-doped Mn_3_O_4_ sample is the maximum. The CV curves of 1.5 at% Cu-doped Mn_3_O_4_ sample with the scan rate ranging from 5 to 100 mV s^−1^ were performed in the potential window of 0 to 0.8 V, as is shown in Fig. 5(b). The CV curves at different scan rates exhibit an approximately rectangular shape, which is an indication of ideal capacitive behavior with excellent reversibility of this electrode material.^32^ The specific capacitance (*C*) was calculated from CV curves according to the following equation:^33^1
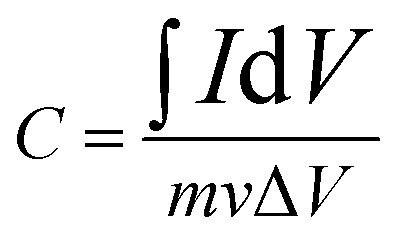
where *I* is the response current, *V* is the potential, *v* is the potential scan rate, *m* is the mass of active electrode material. The specific capacitance is calculated as 194.5 F g^−1^ at a scan rate of 5 mV s^−1^ and 133.3 F g^−1^ at 100 mV s^−1^, which is comparable to the reported values of Mn_3_O_4_ based electrodes in literature.^34–36^ It keeps a comparable high specific capacitance retention even at high scan rate of 100 mV s^−1^, showing a good electrochemical behavior of the device. The energy storage mechanism of Mn_3_O_4_ electrode materials has been accepted as the proton–electron mechanism.^37–39^ While the doped Cu ions may incurs the following redox reactions:^40^ Cu^2+^ + e^−^ ⇌ Cu^+^.

GCD experiments were carried out to test the capacitive performance of the undoped and doped Mn_3_O_4_ samples. The GCD experiments were carried out from 0 to 0.8 V with different current densities from 1 to 10 A g^−1^. The influence of discharge current density on the specific capacity of the undoped and doped Mn_3_O_4_ electrodes was summarized in Fig. 5(c). And Fig. 5(d) shows the representative charge–discharge curve of 1.5 at% Cu-doped Mn_3_O_4_ sample. The ideal charge–discharge characteristic and good reversibility are further confirmed by the linear and symmetric charge–discharge profiles.^41^ The specific capacitance of the electrode was calculated according to the following equation:2
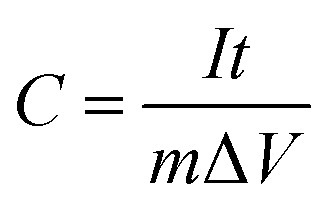
where *I*, *t*, *m* and Δ*V* are constant discharge current (A), discharging time (s), weight (g) of electroactive material and discharging potential range (V), respectively. Using eqn (2), the specific capacitances of undoped, 0.5 at%, 1 at%, 1.5 at% and 2 at% Cu-doped Mn_3_O_4_ samples are calculated to be 180.3, 222.5, 238.2, 257.6 and 169.6 F g^−1^ at the current density of 1 A g^−1^, respectively. The specific capacitance increase with increasing doping concentration of Cu from 0 to 1.5 at%, and the 1.5 at% Cu-doped Mn_3_O_4_ sample shows a maximum specific capacitance of 257.6 F g^−1^ at 1 A g^−1^, a 43% increase in specific capacitance over the undoped one. However, further increase of the Cu content to 2 at% results in a fade electrochemical performance. The specific capacitances have the same order as that of the variation of the CV result with respect to the doping concentration. As can be seen in Fig. 5(c), similar tendency in the specific capacitances for Cu-doped Mn_3_O_4_ and undoped electrodes are maintained at different current densities ranging from 1 to 10 A g^−1^. It clearly shows that the 1.5 at% Cu-doped Mn_3_O_4_ sample depicts the highest specific capacitances among all the prepared samples at all tested current densities, apparently revealing its excellent capacitive performance. The specific capacitance obtained from the discharging curves (Fig. 5(d)) is calculated to be 183.0 F g^−1^ at a current density of 2 A g^−1^, which is almost comparable with the specific capacitance 194.5 F g^−1^ calculated from the CV measurements at a scan rate of 5 mV s^−1^. The improvement in the specific capacitance for 1.5 at% Cu-doped Mn_3_O_4_ sample could be attributed to the bud-like hollow nanostructure as well as the enhanced electrical conductivity, which will be illustrated in following EIS part. The addition of Cu dopants can induce the formation of porous hollow nanostructure with larger surface area and higher pore volume. The mesoporous structures can promote the mass transport of electrolyte within the electrode during the redox reaction process and provide a large number of exposed active sites. Moreover, the direct contact of the deposited material with the underlying conductive Ni foam is highly favorable for electron collection and avoids the use of a polymer binder and conductive additives, and substantially reduces the “dead volume” in the electrode. In conclusion, the higher pore volume of 1.5 at% Cu-doped Mn_3_O_4_ sample may also be useful to facilitate the diffusion of active species and thus contribute to the specific capacitance enhancement.

The cycle stability is a crucial parameter for fast energy storage device. The cycle stability tests of the 1.5 at% Cu-doped Mn_3_O_4_ sample was performed at the current density of 2 A g^−1^ for 6000 times, with undoped Mn_3_O_4_ electrode as reference, as shown in Fig. 5(e). For the 1.5 at% Cu-doped Mn_3_O_4_ sample, it is worth noting that the two curves present a short-termed increase of specific capacitance in the initial hundreds cycles due to an improvement in the surface wetting of the electrode by the electrolyte during extended cycling.^42^ After 6000 charge–discharge cycles, 1.5 at% Cu-doped Mn_3_O_4_ electrode materials show 90.6% of capacitance retention. For undoped Mn_3_O_4_, the specific capacitance presents a slight increase in the initial 750 cycles and thereafter decreased gradually, just 77.4% of initial capacitance can be maintained after 6000 cycles. The results proved that Cu-doped Mn_3_O_4_ materials can be served as promising electrode materials for supercapacitors.

**Fig. 6:**
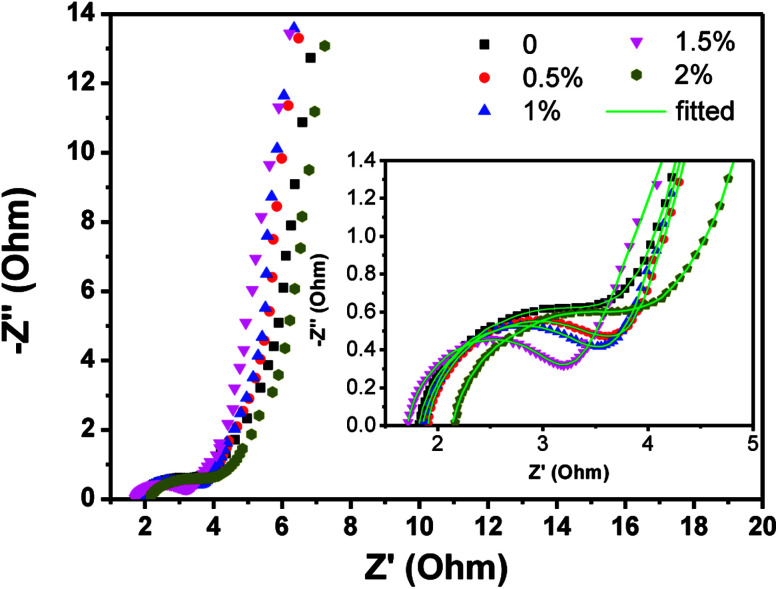
Nyquist impedance spectra of the undoped and Cu-doped Mn_3_O_4_ samples.

The EIS analyses were carried out using an AC voltage of 5 mV in a frequency range from 0.01 Hz to 100 kHz to better understand the electrochemical properties of undoped and Cu-doped Mn_3_O_4_ samples. Fig. 6 shows the Nyquist plots for all samples, two regions of distinct electrochemical response can be seen. At high frequency region, a distorted semicircle is observed; at low frequency, the response is indicative of diffusion process represented by a straight line. It is well known that the power output capability of electrochemical capacitors depends largely upon the equivalent series resistance (ESR).^43^ The ESR, related with the electrode material's conductivity, has been estimated from the *X*-intercept of the Nyquist plot.^26,44^ The ESR values of undoped, 0.5 at%, 1 at%, 1.5 at% and 2 at% Cu-doped Mn_3_O_4_ sample were found to be 1.95, 1.71, 1.62, 1.49 and 1.90 Ω, respectively. The ESR value of samples firstly decreases and then increases with increasing Cu dopant content is clearly observed. The 1.5 at% Cu-doped Mn_3_O_4_ sample exhibits the lowest ESR, which suggested that the electrode has an easy access to ions for the intercalation and deintercalation.^45^ The straight sloping line at low frequency represents the diffusion of ions in the electrode material, and the slopes of these straight sloping lines are different. It was observed that the Nyquist plots of the 1.5 at% Cu doped Mn_3_O_4_ sample displays more steeply rising behavior in the low frequency region as compared to the other samples, which demonstrates that its capacitive performance is much closer to an ideal supercapacitor. Therefore, the highest specific capacitance of the 1.5 at% Cu-doped Mn_3_O_4_ sample can be attributed to the minimum ESR. The above results suggest that the 1.5 at% Cu-doped Mn_3_O_4_ sample is a better candidate for supercapacitor application, as it has been already confirmed by the CV and GCD results.

**Fig. 7:**
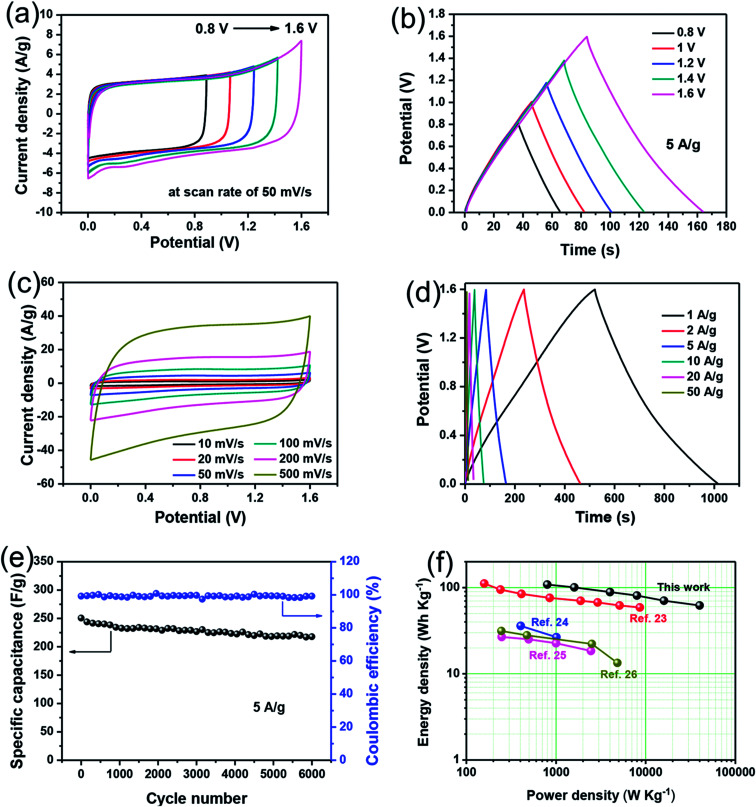
(a) CV curves measured at different potential windows at a scan rate of 50 mV s^−1^, (b) GCD curves in different potential windows at the current density of 5 A g^−1^, (c) CV curves at different scan rates ranging from 10 to 500 mV s^−1^ within the potential window of 0–1.6 V, (d) galvanostatic charge/discharge curves at different current densities, and (e) cycling stability and coulombic efficiency at a constant current density of 5 A g^−1^ of the 1.5 at% Cu-doped Mn_3_O_4_//G ASC. (f) Ragone plots of the 1.5 at% Cu-doped Mn_3_O_4_//G ASC compared with other Mn_3_O_4_ based ASC.

To study the capacitive performance for practical application, the asymmetric supercapacitor (ASC) device was fabricated by 1.5 at% Cu-doped Mn_3_O_4_ sample as the positive electrode, graphene (G) coated Ni foams as the negative electrode, polytetrafluoroethylene (PTFE) membrane separator and 1 M Na_2_SO_4_ aqueous solution as the electrolyte. This assembled ASC was denoted as Mn_3_O_4_ (1.5Cu)//G. To obtain the maximum performance of the Mn_3_O_4_ (1.5Cu)//G ASC, it is crucial to keep the charges balanceable with the relationship *q*^+^ = *q*^−^. In order to get the charge balanceable, the optimum loading mass of AC was decided by the following equation:

3 *m*_(Mn_3_O_4_(1.5Cu))_/*m*_(G)_ = *C*_m(G)_Δ*V*_(G)_/*C*_m(Mn_3_O_4_(1.5Cu))_Δ*V*_(Mn_3_O_4_(1.5Cu))_

where *m* is the mass of activated material, *C*_m_ represents the specific capacitance and Δ*V* is the potential window in the three-electrode test system. The GDC curve of the G electrode at 1 A g^−1^ is depicted in Fig. S2† and the calculated *C*_m_ value of the G electrode is 171.7 F g^−1^. The *C*_m_ value of the Mn_3_O_4_ (1.5Cu) electrode is 257.6 F g^−1^ at 1 A g^−1^ (Fig. 5(c)). The optimal mass loading of G in Mn_3_O_4_ (1.5Cu)//G was 3 mg. Fig. 7(a) displays the CV curves of the 1.5 at% Cu-doped Mn_3_O_4_//G ASC in various potential windows (0.8–1.6 V) recorded at a constant scan rate of 50 mV s^−1^. They reveal that the 1.6 V potential is suitable for the 1.5 at% Cu-doped Mn_3_O_4_//G ASC as it shows a higher area under the CV curve and better current response. The GCD curves of 1.5 at% Cu-doped Mn_3_O_4_//G ASC exhibit symmetrical triangular shapes in the different potential windows (0.8 to 1.6 V) at the current density of 5 A g^−1^ (Fig. 7(b)), further indicating an ideal capacitive behaviour. Therefore, the 1.6 V potential was selected as the suitable operational voltage range for further electrochemical measurements. Fig. 7(c) shows the CV curves of the assembled ASC at different scan rates ranging from 10 to 500 mV s^−1^ within the potential window of 0–1.6 V. The shape of CV curves can be well retentive with increasing the scan rates, suggesting the ideal electron-transfer kinetics and good rate capability of the asymmetric supercapacitor. Fig. 7(d) shows the typical GCD curves of the ASC measured at various current densities in a potential window of 0–1.6 V. The GCD curves at various current densities from 1 A g^−1^ to 50 A g^−1^ manifest approximately symmetrical triangular shapes, demonstrating a good reversibility between the charge and discharge processes.^46^ The specific capacitance calculated from galvanostatic discharge curves of the ASC device in Fig. 7(d) is 305.6 F g^−1^ at 1 A g^−1^. Even at a high current density of 50 A g^−1^, the specific capacity remains at 175.0 F g^−1^ (about 57.3% of the capacitance retained), showing the good rate capability.

Long-term cycling performance is also one of important factors for supercapacitor applications. The cycling stability of the 1.5 at% Cu-doped Mn_3_O_4_//G ASC is evaluated by the repeated charging/discharging measurement at a constant current densities of 5 A g^−1^ between 0 and 1.6 V. As shown in Fig. 7(e), the specific capacitance of the device decreases gradually with increasing cycling times, and still retains 86.9% of the initial specific capacitance even after 6000 cycles. Meanwhile, its coulombic efficiency maintained high values over 99% at the whole cycles. These results suggest that the 1.5 at% Cu-doped Mn_3_O_4_//G ASC delivers good electrochemical stability. Moreover, to evaluate the energy storage ability of the 1.5 at% Cu-doped Mn_3_O_4_//G ASC, the Ragone plots based on the galvanostatic discharge curves are displayed in Fig. 7(f). The energy and power density of the ASC devices were calculated based on these equations:^47^

4 *E* = *C*_m_Δ*V*^2^/2

5 *P* = *E*/Δ*t*

where *E* (W h kg^−1^) is the average energy density; *C*_m_ (F g^−1^) is the specific capacitance of the ASC device; Δ*V* (V) is the voltage window; *P* (W kg^−1^) is the average power density and Δ*t* (s) is the discharge time. As shown in Fig. 7(f), the ASC device could achieve a high *E* value of 108.6 W h kg^−1^ (with a *P* value of 799.9 W kg^−1^) and still maintain 62.2 W h kg^−1^ at a high power density of 39 993.8 W kg^−1^, which is superior to many previously reported Mn_3_O_4_-based ASCs, such as such as Mn_3_O_4_/rGO//GO ASC,^48^ Mn_3_O_4_/AC based ASC,^49^ Mn_3_O_4_/rGO nanocomposite based ASC,^50^ and Mn_3_O_4_–GO double-shell hollow sphere based ASC.^51^ The superior energy density of the fabricated 1.5 at% Cu-doped Mn_3_O_4_//G ASC device can be attributed to its high specific capacitance and wide potential window. Therefore the 1.5 at% Cu-doped Mn_3_O_4_ could be a promising electrode material for a high performance supercapacitor.

## 4. Conclusion

In summary, we have successfully fabricated hierarchical Cu-doped Mn_3_O_4_ hollow nanostructures on Ni foam by a simple hydrothermal method. The existence of Cu can not only induce the formation of the unique bud-like hollow nanostructure, but also effectively increase the conductivity of the electrode. It was found that Cu doping plays a promotional role in enhancing Mn_3_O_4_ behavior in electrochemical capacitors. Electrochemical test confirms that 1.5 at% Cu-doped Mn_3_O_4_ could achieve a high specific capacitance (257.6 F g^−1^ at 1 A g^−1^) and long-term cycling stability (90.6% capacitance retention after 6000 times repetition at current density of 2 A g^−1^), holding a promise to be applied in high-performance asymmetric supercapacitor. Meanwhile, a 1.5 at% Cu-doped Mn_3_O_4_//G ASC is further fabricated and exhibits a good electrochemical performance with high specific capacitance, excellent long-term cycling stability, and good electrochemical reversibility of the faradaic reaction. As a result, the obtained bud-like Cu-doped Mn_3_O_4_ can be a promising electrode material for future energy storage devices.

## Conflicts of interest

The authors declare no conflict of interest.

## Supplementary Material

RA-008-C8RA06989A-s001
