# Supplementary material for: Performance enhancement of asymmetric supercapacitors with bud-like Cu-doped Mn3O4 hollow and porous structures on nickel foam as positive electrodes
Source: RSC Adv. 2018 Oct 22;8(63):35878–87. doi: 10.1039/c8ra06989a (PMC9088714; doi:10.1039/c8ra06989a)
Supplement: RA-008-C8RA06989A-s001 [file RA-008-C8RA06989A-s001.pdf]

Electronic Supplementary Material (ESI) for RSC Advances.
This journal is © The Royal Society of Chemistry 2018

Supporting Information

Performance enhancement of asymmetric supercapacitor with bud-like Cu-doped Mn_3O_4 hollow and porous structures on nickel foam as positive electrode

Xiaobo Chen,[a] Cheng Chen,[a] Tianzhi Xu,[a] Yingjie Xu,[a] Weiwei Liu,[a] Wen Yang,[b] Peizhi Yang[*b]

[a]School of New Energy and Electronic Engineering, Yancheng Teachers University, Yancheng, 224051, PR China

[b]Key Laboratory of Education Ministry for Advance Technique and Preparation of Renewable Energy Materials, Yunnan Normal University, Kunming, 650500, PR China

E-mail addresses: pzhyang@hotmail.com (P. Yang)

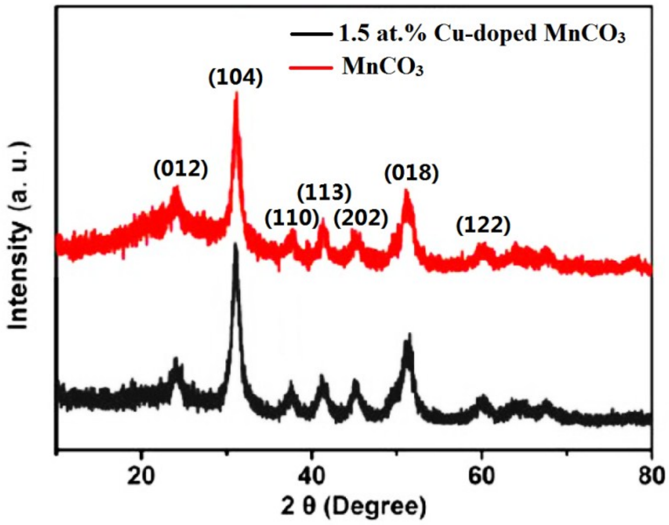

Fig.S1. XRD patterns of the samples $MnCO_3$ and 1.5 at.% Cu-doped $MnCO_3$.

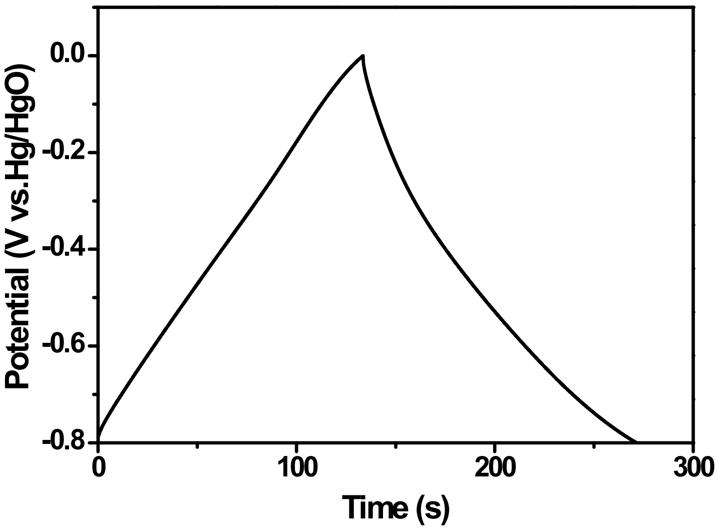

Fig.S2. Galvanostatic charge–discharge curve of the G electrode at a current density of 1 A g^{-1}.
